# EGCG Promotes Neurite Outgrowth through the Integrin *β*1/FAK/p38 Signaling Pathway after Subarachnoid Hemorrhage

**DOI:** 10.1155/2021/8810414

**Published:** 2021-01-25

**Authors:** Yuyuan Zhang, Mengguo Han, Xiaoxue Sun, Guojun Gao, Guoying Yu, Liong Huang, Ying Chen

**Affiliations:** ^1^College of Life Sciences, Henan Normal University, Xinxiang, China; ^2^Department of Neurosurgery, The First Affiliated Hospital of Xinxiang Medical University, Weihui, Henan 453100, China; ^3^Institute of Biomedicine, Henan Normal University, Xinxiang, China

## Abstract

The abnormal neurites have long been regarded as the main player contributing to the poor outcome of patients with subarachnoid hemorrhage (SAH). (-)-Eigallocatechin-3-gallate (EGCG), the major biological component of tea catechin, exhibited strong neuroprotective effects against central nervous system diseases; however, the role of EGCG-mediated neurite outgrowth after SAH has not been delineated. Here, the effect of reactive oxygen species (ROS)/integrin *β*1/FAK/p38 pathway on neurite outgrowth was investigated. As expected, oxyhemoglobin- (OxyHb-) induced excessive ROS level was significantly reduced by EGCG as well as antioxidant N-acetyl-l-cysteine (NAC). Consequently, the expression of integrin *β*1 was significantly inhibited by EGCG and NAC. Meanwhile, EGCG significantly inhibited the overexpression of phosphorylated FAK and p38 to basal level after SAH. As a result, the abnormal neurites and cell injury were rescued by EGCG, which eventually increased energy generation and neurological score after SAH. These results suggested that EGCG promoted neurite outgrowth after SAH by inhibition of ROS/integrin *β*1/FAK/p38 signaling pathway. Therefore, EGCG might be a new pharmacological agent that targets neurite outgrowth in SAH therapy.

## 1. Introduction

A growing body of epidemiological and animal evidence demonstrated that green tea dramatically lowered the incidence of strokes and Alzheimer's disease risk. Moreover, people who drank more than two cups of green tea per day exhibited less cognitive impairment after neuronal injury [1, 2]. The main components of green tea include (-)-epigallocatechin (EGC), (-)-epicatechin-3-gallate (ECG), (-)-epicatechin (EC), and (-)-epigallocatechin-3-gallate (EGCG). Notably, EGCG played pivotal roles in the prevention and treatment of almost all diseases largely dependent on its well-known anti-oxidative activity [3, 4]. Besides, we previously reported that EGCG exhibited strong neuroprotective effects against subarachnoid hemorrhage (SAH) by inhibition of oxidative stress, Ca^2+^ overloading, mitochondrial dysfunction, and cell death [5–8].

Being a devastating subtype of strokes, the mortality rate of SAH can be as high as 67% [9]. More importantly, it has been estimated that 36–55% survivors suffered impairment, disability, handicap, and poor quality of life during the first year after SAH, which might predict functional outcome of neuronal injury [10]. Neuritis, composed of axon and dendrites, is the structural unit determining the function of the brain [11]. Contrary to the reversible axonotmesis in myelinated and unmyelinated white matter, neurite degeneration might be less likely to be rescued in gray matter, which is mainly made up of neuronal cell bodies and dendrites. Therefore, abnormal neurite morphology and length have been regarded as a common factor contributing to aging, neurodegenerative diseases, and central nervous system (CNS) injury [12–14]. Recently, multifocal axonal injury has also been observed in the early stage after SAH. Most importantly, axonal degeneration has been a major player determining primary injury and the outcome of SAH patients. Few molecules, such as ROS, Ca^2+^, neurofilament high chain (NfH), and S100, have been identified regulating axonal damage after SAH. However, the precise mechanism of axonal degeneration after SAH has not been fully understood.

EGCG has been demonstrated to regulate nerve growth factor- (NGF-) induced neurite outgrowth [15]. It has been reported that EGCG bound to 67 LR and inhibited ROS generation to promoting axonal regeneration. However, whether EGCG induces neurite outgrowth after SAH and the underlying mechanisms remain unclear. Many intracellular signaling pathways have been identified to regulate neurite outgrowth, of which the integrin family of adhesion molecules played pivotal roles and might be regarded as important therapeutic targets by activating “outside-in” signaling molecules, such as FAK, ERK, p38, and JNK [16–18]. In our previous study, EGCG (50 *μ*M *in vitro* or 50 mg/kg *in vivo*) significantly inhibited ROS generation and the overexpression of p38 and eventually rescued neuronal cells injury after SAH. Based on these findings, we aimed to investigate the neuroprotective effects of EGCG on neurite outgrowth via ROS/integrin *β*1/FAK/p38 pathway by using SAH cell and mice models [5–8].

## 2. Materials and Methods

### 2.1. Materials

PC12 cells were bought from the Cell Bank of Chinese Academy of Sciences (Shanghai, China). EGCG with a 95% purity (Huike, Shanxi, China) was diluted by water (pH 3.0) and stored at −20°C. NGF was gained from Sigma (St. Louis, MO, USA). OxyHb was obtained from Shanghai Sangon Biotech (China). FAK inhibitor Y15 and p38 inhibitor SB203580 were obtained from Selleck (Houston, TX, USA). Primary antibodies against integrin beta 1, pFAK, and p-p38 (Thr180/Tyr182) were purchased from Cell Signaling Technologies (Danvers, MA, USA). Penicillin and streptomycin, N-Acetyl-L-cysteine (NAC), and an enhanced chemiluminescence (ECL) kit were purchased from Beyotime (Shanghai, China). ATP and lactate dehydrogenase (LDH) assay kit were obtained from Nanjing Jiancheng Bioengineering Institute (Jiangshu, China). The First-Strand Complementary DNA (cDNA) Synthesis Kit (11483188001) was purchased from Roche Applied Sciences (Basel, Switzerland). Power SYBR® Green PCR Master Mix was obtained from Thermo Fisher (Waltham, Massachusetts, USA).

### 2.2. Cell Culture and Treatment

The cells were cultured at 37°h in 5% CO_2_ in RPMI 1640 medium supplemented with 1% fetal bovine serum (Thermo Fisher Scientific, Waltham, MA, USA), 1% penicillin/streptomycin, and 50 ng/ml NGF for 24 h. Then, OxyHb was added to the medium to a final concentration of 10 *μ*M. EGCG (50 *μ*M) was added 30 min before the addition of OxyHb as EGCG pretreatment group [7]. NAC (2.5 *μ*M) and p38 inhibitor (20 *μ*M) were added 30 min after the addition of OxyHb as NAC and p38 inhibitor treatment groups [19].

### 2.3. Animals

The animal use and care protocols were approved by the Institutional Animal Care and Use Committee (IACUC) of Henan Normal University. Male Kunming mice (*n* = 150, weighing from 20 to 25 g) were bought from Zhengzhou University. The animals were kept in a constant temperature (22 ± 3°C) and relative humidity (60%) animal rooms in a 12 h light/dark cycle. All animals were allowed to undergo appropriate quarantine procedures.

### 2.4. Mouse SAH Model

SAH mice model was established based on what was previously reported by Huang et al. [20]. After being given 1% pentobarbital general anesthesia (50 mg/kg, intraperitoneally), the posterior cervical muscles of mice were dissected through midline suboccipital approach to penetrate the transparent atlanto-occipital membrane. Then, a hole was made in the cross position at 2 mm from the sagittal suture and 1 mm from the coronaria suture. Subsequently, 30 *μ*L of OxyHb (150 *μ*mol/L) was injected into the subarachnoid space, whereas the isotonic saline was administered as the sham group. As for EGCG treatment group, EGCG was intragastrically administered at a dose of 50 mg/kg/d for 14 days before OxyHb injection. As for NAC, FAK inhibitor, and p38 inhibitor treated groups, a total of NAC, Y15, and SB203580 (150 mg/kg, 30 mg/kg, and 200 *μ*g/kg, respectively) was injected into subarachnoid space after 30 min of SAH established [20]. The mice were sacrificed and brain tissue was collected after 48h of SAH.

### 2.5. Experimental Design

#### 2.5.1. Experimental Design for Exploring the Roles of Superoxide Anion and Neurite Outgrowth in *In Vitro* SAH Model

NGF-induced PC12 were divided into 4 groups to investigate superoxide anion concentration (control, SAH, SAH + EGCG, SAH + NAC) and the changes of neurite outgrowth (control, SAH, SAH + EGCG, SAH + p38 inhibitor).

#### 2.5.2. Experimental Design for Exploring the Roles of Integrin Beta 1, FAK, and p38 in *In Vivo* SAH Model

In this experiment, mice (*n* = 150) were randomly divided into the following 6 groups: control, SAH, SAH + EGCG, SAH + NAC, SAH + FAK inhibitor, and SAH + p38 inhibitor:

  The four groups (*n* = 10 per group) (control, SAH, SAH + EGCG, and SAH + NAC) were used to detect the expression of integrin.
  The four groups (*n* = 10 per group) (control, SAH, SAH + EGCG, and SAH + p38 inhibitor) were used to detect the expression of pFAK.
  The four groups (*n* = 10 per group) (control, SAH, SAH + EGCG, and SAH + FAK inhibitor) were used to detect the expression of p-p38.
  The three groups (*n* = 10 per group) (control, SAH, and SAH + EGCG) were used to detect ATP, LDH, and neurological score.

### 2.6. Measurement of Superoxide Anion Levels

The cell permeable non-fluorescent DHE was used to detect the intracellular superoxide anion levels according to the manufacturer's instruction. After the experiment, serum-free culture medium was used to wash the cells. Then, DHE probe was added to a final concentration of 10 *μ*M and incubated at 37°C for 15 min. The fluorescence was measured by a microplate reader using 300 nm excitation and 610 nm emission wavelength.

### 2.7. Fluorescence Microscopy

The cells were seeded on glass coverslips. After washing with PBS, the cells were incubated with DHE (10 *μ*M) for 15 min as described previously [8]. Fluorescence signals were visualized with a Delta Vision Spectris fluorescence microscopy (AX10, Zeiss).

### 2.8. Measurement of Neurite Outgrowth

To study neurite outgrowth, the total length of neurites and neurite outgrowth cells were observed by microscope (Zeiss Oberkochen, Germany). The length of neurites was measured using Imaging software Presage (Brooksville, FL, USA). The cell was counted as a neurite outgrowth cell when the length was equal to or greater than the diameter of the cell body. At least, 50 cells were measured and all experiments were performed in triplicate three times.

### 2.9. Neurological Functions Assessment

Animal neurological behavior and function were evaluated using the Garcia scoring system [21]. Briefly, the neurobehavioral examination was performed at 48 h. An 18-point scoring system was used based on (1) spontaneous activity, (2) symmetry of limb movement, (3) climbing, (4) body proprioception, (5) the movement of forelimbs, and (6) response to vibrissae touch.

### 2.10. Brain Cortex ATP Content Assessment

Boiling double distilled water was used to homogenize cerebral cortex to denature endogenous ATPase. After being centrifugated at 10,000 g for 10 min, the supernatant fraction was collected. ATP levels were measured according to manufacturer's instructions.

### 2.11. Lactate Dehydrogenase (LDH) Assay from Brain Cortex

The supernatant fraction of all the samples was collected to detect LDH activity according to the manufacturer's instructions. LDH cytotoxicity was calculated using OD as LDH cytotoxicity (U/g protein) = (OD sample – OD blank)/(OD standard solution – OD blank standard solution) × standard solution concentration/sample protein concentration.

### 2.12. Western Blot Assay

Brain cortex was homogenized (1% proteinase K inhibitor, PMSF 1 mM, and 1% (v/v) protease inhibitor cocktail) and centrifugated at 10000 g for 10 min at 4°C. The collected supernatant was frozen at −80°C. Total protein (50 *μ*g) from each group was loaded and separated by 12% SDS-PAGE and electro-transferred to nitrocellulose membrane. After being blocked with non-fat milk in PBS for 1 h, the membrane was incubated with monoclonal antibody against integrin beta 1 (1 : 1000), pFAK (1 : 500), and GAPDH (1 : 400) overnight, respectively. Being washed three times with PBS containing 0.05% Tween-20, the membrane was incubated with a secondary antibody for 1 h. The densities of the bands were visualized and analyzed by using enhanced chemiluminescence (ECL) kit and ImageQuant software. All the experiments were independently repeated three times.

### 2.13. Quantification of mRNA Expression of Integrin Genes Using RT-PCR

qRT-PCR was carried out based on our previous report [7]. Briefly, the total RNA was isolated and each RNA sample was reverse-transcribed. Then, qRT-PCR was performed to identify the mRNA expression of integrin beta 1 (NM_017022.2) and integrin beta 5 (NM_147139.2) by using the BIO-RAD CFX Connect^TM^ platform (Hercules, California, USA). Briefly, qRT-PCR reactions were performed in a total volume of 25 *μ*l containing 2.5 *μ*l of cDNA, 0.5 *μ*l of primes (5 *μ*M), 12.5 *μ*l of PCR Master Mix, and 9.5 *μ*l of deionized water. GAPDH was used to normalize each target gene by using the comparative 2^−ΔΔCt^ method. The sequences of mRNA primers were listed as follows:

Integrin beta 1: Fw: 5′-ACAGAAGAAGTAGAGGTGGTCC-3′,
    Rw: 5′-GCTACATTCACAGTGTCTCCC-3′;
Integrin beta 5: Fw: 5′-TTGCGGAGGAGATGAGGAAG-3′,
    Rw: 5′-AAGGGACACAGTTGGGGAAT-3′;
GAPDH: Fw: 5′-TGACTCTACCCACGGCAAG-3′,
    Rw: 5′-ACTCAGCACCAGCATCACC-3′.

### 2.14. Immunohistochemistry Assay

Deparaffinized sections were heated in sodium citrate buffer (0.01 M, pH 6.5) for 10 min at 121°C. Subsequently, sections were incubated with 0.3% hydrogen peroxide to block endogenous peroxidase activity. After being blocked with 10% normal goat serum, primary anti-p-p38 antibody was used at a 1 : 1000 dilution and incubated overnight. After further washing, slides were treated with Histostain^™^-Plus kit (SP-9001, Zymed, USA). Immunoperoxidase staining was developed using DAB (ZLI-9032, Zhongshan Golden Bridge Biotechnology Co., Ltd., China). At least 10 visual fields were captured and more than 500 cells were counted using the IDA-2000 software (Beijing Konghai Technology Company, China).

### 2.15. Statistical Analysis

Data were expressed as means ± SD. Significant differences were verified with a one-way ANOVA analysis, followed by Tukey's test for multiple comparisons by using SPSS 17.0 software. Kruskal–Wallis nonparametric test followed by Duncan's multiple-comparison procedures was used for the neurological scores. *p* < 0.05 was considered as statistically significant.

## 3. Results

### 3.1. EGCG Inhibited Superoxide Anion Generation after SAH

**Figure 1:**
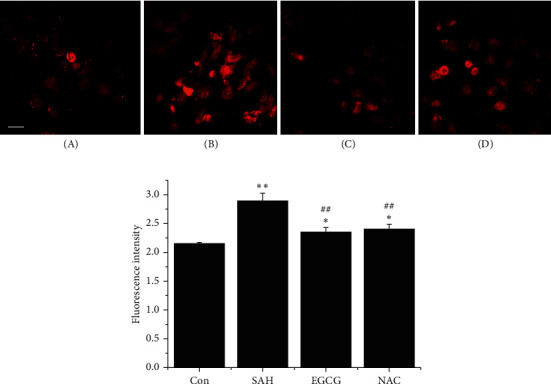
EGCG inhibited superoxide anion generation after SAH. (a) Superoxide anion fluorescence distribution in cells detected by fluorescence microscopy. Normal cells showed less fluorescence (A). OxyHb at a final concentration of 10 *μ*M significantly increased fluorescence intensity (B). EGCG, added 30 min before the addition of OxyHb, significantly reduced superoxide anion level almost to basal level (C). Similar trend of superoxide anion level was also observed in NAC group, which was added 30 min after the addition of OxyHb (D). Bar, 200 *μ*m. (b) Quantification of superoxide anion fluorescence intensity. The values represent three independent experiments. ^*∗*^*p* < 0.05 vs. control, ^*∗∗*^*p* < 0.01 vs. control, and ^##^*p* < 0.01 vs. SAH.

OxyHb-induced oxidative stress has long been regarded as an early event in the progression of SAH. DHE is oxidized by superoxide anion to form ethidium, which binds to DNA. Therefore, more red fluorescence indicated higher superoxide anion levels. Compared with the control group (2.2 ± 0.2), strong signals were captured in SAH group showing an increase in red fluorescence intensity (2.9 ± 0.1, *p* < 0.01 vs. control), whereas less signals were detected in the EGCG and NAC treatment group (*p* < 0.01 vs. SAH) (Figure 1).

### 3.2. EGCG Downregulated Integrin *β*1 Expression through ROS

**Figure 2:**
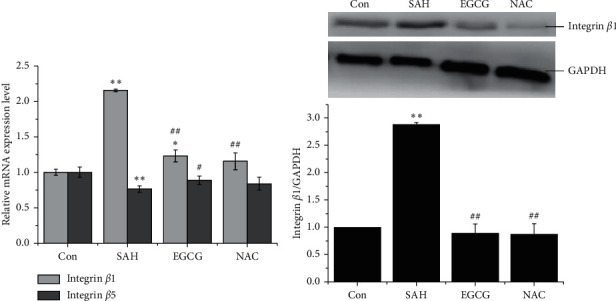
EGCG inhibited the overexpression of integrin *β*1 after SAH. (a) EGCG regulated integrin *β*1 and integrin *β*5 mRNA expression level in *in vivo* SAH model. (b) Identification of integrin *β*1 protein level in *in vivo* SAH model (upper panel) (*n* = 3), and quantification of the expression of integrin *β*1 (lower panel). SAH: 30 *μ*L of OxyHb (150 *μ*mol/L); EGCG: 50 mg/kg/d; NAC: 150 mg/kg. A representative trace of three repeats of each experiment is shown. ^*∗*^*p* < 0.055 vs. control, ^*∗∗*^*p* < 0.01 vs. control, ^#^*p* < 0.01 vs. SAH, and ^##^*p* < 0.01 vs. SAH.

Given that ROS functioned as an inducer to activate integrins, the expression of integrins *β*1 and *β*5 was measured after SAH. Contrary to the high ROS concentrations in SAH group, the downregulated mRNA expression of integrin *β*5 was observed (*p* < 0.01 vs. control), whereas the expression of integrin *β*1 was significantly increased 1.25-fold after SAH (*p* < 0.01 vs. control) (Figure 2(a)). However, EGCG significantly restored the imbalance in the expression of integrin *β*5 (*p* < 0.05 vs. SAH) to basal level but not integrin *β*1 (*p* < 0.05 vs. control, *p* < 0.01 vs. SAH). NAC has the similar anti-oxidative capacity in regulation of the elevated expression of integrin *β*1 (*p* < 0.01 vs. SAH) and *β*5 (*p* > 0.05 vs. SAH). Notably, the protein expression level of integrin *β*1 is similar to the mRNA expression trend, showing that both EGCG and NAC significantly inhibited the overexpression of integrin *β*1 after OxyHb stimuli (*p* < 0.01 vs. SAH), demonstrating that ROS triggered the overexpression of integrin *β*1 after SAH (Figure 2(b)).

### 3.3. EGCG Inhibited FAK and p38 Signaling after SAH

**Figure 3:**
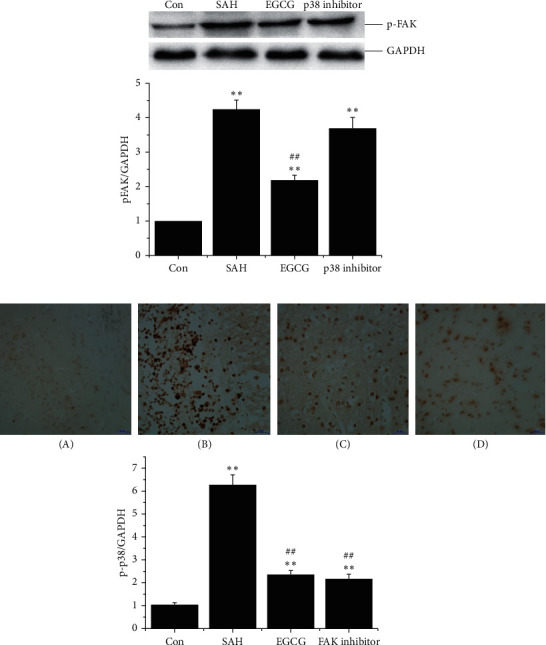
The expression of pFAK and p-p38 after SAH *in vivo*. (a) EGCG downregulated the expression of pFAK (upper panel) (*n* = 3). SAH: 30 *μ*L of OxyHb (150 *μ*mol/L); EGCG: 50 mg/kg/d; p38 inhibitor: 200 *μ*g/kg. Quantification of the expression of pFAK (lower panel). (b) EGCG inhibited p-p38 overexpression after SAH (upper panel) (*n* = 3) (bar, 50 *μ*m), and quantification of the expression of p-p38 (lower panel). (A) control; (B) SAH (30 *μ*L (150 *μ*mol/L) of OxyHb); (C) EGCG (50 mg/kg/d); (D) FAK inhibitor (30 mg/kg). A representative trace of three repeats of each experiment is shown. ^*∗∗*^*p* < 0.01 vs. control and ^##^*p* < 0.01 vs. SAH.

To elucidate the mechanism through which EGCG modulated integrin signaling pathway, the downstream effectors, FAK and p38, were examined. In line with the finding of integrin *β*1, the expression of phosphorylated FAK and p38 significantly reached 4.2 ± 0.3 and 6.3 ± 0.5 in SAH group (*p* < 0.01 vs. control), whereas EGCG dramatically downregulated the expression of FAK and p38 phosphorylation (2.2 ± 0.2 and 2.4 ± 0.2, respectively, *p* < 0.01 vs. SAH). More importantly, FAK inhibitor significantly antagonized the expression of phosphorylated p38 after SAH, whereas the expression of pFAK showed no significant difference between p38 inhibitor and SAH group, indicating that p38 might be the downstream effector of FAK in response to integrin *β*1 overexpression after OxyHb stimuli (Figures 3(a) and 3(b)).

### 3.4. EGCG Promoted Neurite Outgrowth by Inhibition of Excessive Activation of Integrin *β*1/FAK/p38 Pathway

**Figure 4:**
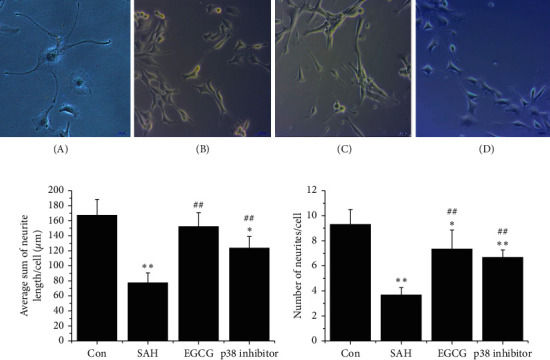
EGCG induced PC12 cells neurite outgrowth. (a) The morphology of NGF-induced PC12 cells after SAH *in vitro*. (A) Control; (B SAH (at a final concentration of 10 *μ*M OxyHb; (C) EGCG (50 *μ*M); (D) NAC (2.5 *μ*M). Bar, 200 *μ*m for (A) and 100 *μ*m for (B–D). (b) The length of neurites after SAH. (c) The average number of neurites per cell after SAH. A representative trace of three repeats of each experiment is shown. ^*∗*^*p* < 0.05 vs. control, ^*∗∗*^*p* < 0.01 vs. control, and ^##^*p* < 0.01 vs. SAH.

To examine the effect of EGCG on neurite outgrowth after SAH, the NGF-induced PC12 cells were used to observe the length of neurites and number of neurite cells. As shown in Figure 4(a), OxyHb impaired the neuritogenic action. The length of neurites and the number of neurite cells significantly decreased from 168.6 ± 21.1 and 9.3 ± 1.2 in control group to 78.1 ± 12.3 and 3.7 ± 0.6 in SAH group (*p* < 0.01). However, after EGCG treatment, the length of neurites was significantly increased and more neurite cells were observed (152.7 ± 18.9, *p* < 0.01 vs. SAH; 7.3 ± 1.5, *p* < 0.01 vs. SAH), suggesting that neurite outgrowth was induced by EGCG (Figures 4(b) and 4(c)). Given that integrin *β*1/FAK/p38 pathway was involved in NGF-induced neurite outgrowth, we next examined the effects of the specific p38 inhibitor on neuritogenesis. As expected, SB203580 significantly promoted neurite outgrowth after SAH as well as the number of neurite cells (124.9 ± 15.2, 6.7 ± 0.6) (Figure 4).

### 3.5. EGCG Protects Neurological Function after SAH

**Figure 5:**
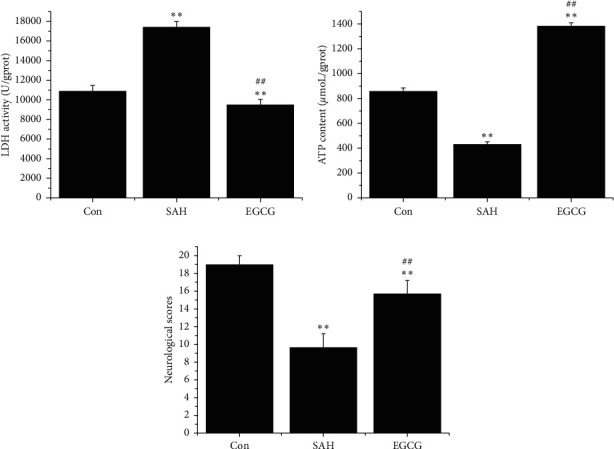
Neuroprotective effects of EGCG after SAH. (a) Effect of EGCG on LDH activity after SAH (*n* = 3). (b) EGCG increased ATP content after SAH (*n* = 3). (c) Neurological deficits after SAH (*n* = 6). SAH: 30 *μ*L of OxyHb (150 *μ*mol/L); EGCG: 50 mg/kg/d. A representative trace of three repeats of each experiment is shown. ^*∗∗*^*p* < 0.01 vs. control; ^##^*p* < 0.01 vs. SAH.

LDH activity is the most widely used marker in cytotoxic studies. Using this assay, we detected the neuroprotective role of EGCG against SAH. LDH activity was lower in the cortical tissue of the control group (10909.0 ± 550.2) (Figure 5(a)). After SAH, LDH levels significantly increased to 17379.6 ± 602.8 (*p* < 0.01 vs. control). However, EGCG induced a dramatic decrease in LDH activity (9424.1 ± 595.4, *p* < 0.01 vs. SAH).

Similar to the finding of LDH, the ATP level was significantly decreased from 859.4 ± 23.5 in the control group to 432.9 ± 18.3 in the SAH group (*p* < 0.01 vs. control), whereas EGCG restored ATP level to basal level (1382.1 ± 26.2, *p* < 0.01 vs. control, *p* < 0.01 vs. SAH), suggesting that EGCG significantly increased ATP content after SAH (Figure 5(b)).

Following the higher energy generation and lower toxicity in the EGCG treatment, the preconditioned EGCG animals showed improved neurological scores (*p* < 0.01 vs. SAH) contrary to severe behavioral disabilities after SAH (*p* < 0.01 vs. control), indicating that impaired behavioral function caused by SAH could be restored by EGCG (Figure 5(c)).

## 4. Discussion

Neuronal cells injury, particularly for the detected axonal degeneration, has been demonstrated to be the major player contributing to the outcome of SAH patients by leading to irreversible neurological deficit [22–24]. Mounting evidence has demonstrated the positive neuroprotective role of EGCG in regulating neurite outgrowth [15, 25]. However, few studies have focused on the mechanisms of EGCG against neurite degeneration after SAH. In the present study, *in vitro* and *in vivo* SAH models were used to investigate the mechanism through which EGCG promoted neurite outgrowth and associated signaling pathway.

Neuronal cells are especially vulnerable to oxidative damage, partly because of the high level of oxygen consumption and the low level of ROS scavenging ability. After SAH, OxyHb oxidized to methaemoglobin (MetHb), which induced ROS generation. Given that oxidative stress was positively associated with the poor outcome of SAH patients, antioxidant therapy has long been regarded as a promising strategy promoting neurons survival under the harmful conditions [26, 27]. Even though EGCG playes a dual role in its physiologicol activities, i.e., the harmful pro-oxidative (high-dose) and the benificial antioxidative activities (low-dose), most studies have, recently, reported that the harmful effect of EGCG was overwhelmed by its benificial role under pathological conditions [28]. These findings were supported by our previous studies showing that EGCG exhibited neuroprotective effects by inhibition of mitochondrial dysfunction-induced oxygen stress after SAH.

Several research groups reported that ROS activated integrin-mediated “outside-in” signaling pathways, whereas others found that integrin, in turn, induced ROS generation and activated “inside-out” signaling [29]. Integrins are heterodimeric membrane adhesion molecules composed of noncovalently bound *α*- and *β*-subunits, of which integrin *β*1 is widely expressed in the brain and involved in morphogenesis, cell differentiation, and proliferation as well as cell survival. The overexpression of integrin *β*1 and FAK aggravated neurological deficit in Alzheimer's disease (AD) and cerebral ischemia-reperfusion [30, 31]. However, after intracerebral hemorrhage, increased expression of integrin *β*1 in the brain significantly improved neurobehavioural score with decrease of neutrophil infiltration through JAK2/STAT1 pathway [32]. Besides, a growing body of evidence showed that integrin *β*1 modulated neuronal migration to regulate neurovascular remodeling, brain edema, and functional outcomes following stroke [33–36]. Therefore, *β*1 integrin has been regarded as a potential target in strokes therapy. Unfortunately, even though there are a few studies about the role of integrin after SAH, inconsistent results have been reported. The expression of integrin *β*3 reduced oxidative stress to inhibit cell death after SAH, whereas the overexpression of GPIIB/IIa integrin has also been reported inducing cell death and neurobehavioral injury in early brain injury after SAH [37–39]. Given that EGCG has been demonstrated to regulate the expression of *α*IIb, *α*5, *β*1, and *β*3 integrins, in the present study, we focused on the role of integrin *β*1 after EGCG treatment of SAH and demonstrated that EGCG inhibited ROS-induced overexpression of integrin *β*1 after SAH [37, 40]. FAK is a well-known downstream effector of ROS-integrin signaling pathway under oxidative stress condition. Inactivation of FAK in mouse endothelium caused brain hemorrhage, and several researches reported that increased phosphorylated FAK resulted in the attenuation of neuronal cell death and improved neurological status after SAH [41, 42]. However, we observed a significant increase in the expression of FAK phosphorylation, whereas EGCG restored the expression of phosphorylated FAK to basal level, indicating the higher expression of phosphorylated FAK and the severe pathological state after SAH. Recently, several substrates of integrin *β*1/FAK have been identified in the SAH progression, such as p38 [43]. FAK inhibitor significantly inhibited the expression of p38, whereas the expression of FAK was maintained at the same level after p38 inhibitor treatment, indicating that p38 was the downstream signal molecule of FAK. In addition, the overexpression of p38 has been reported to induce mitochondrial dysfunction, oxidative stress, apoptosis, and autophagy after SAH. Notably, EGCG inhibited the overexpression of p-p38 against mitochondrial dysfunction and cell death after SAH [5, 20]. Therefore, the neuroprotective role of EGCG partly relied on integrin *β*1/FAK/p38 signaling pathway after SAH [44].

Under serum-free condition, NGF induced neurite outgrowth of PC12 cells. This well-defined neuronal model is often employed for studying neurotoxicity in some CNS diseases. Integrins affect neurite outgrowth in adult healthy brains and pathological conditions [45–47]. Farizatto et al. found that the overexpression of integrin *β*1 regulated synapse maintenance and plasticity [48]. However, some research showed that the lower level of integrin *β*1 reduced dendrite growth, suggesting that integrin *β*1/FAK signaling pathway was involved in neurite outgrowth, which contributed to the differentiation of neuronal cells and determined the outcome of patients [31, 49]. In the present study, EGCG dramatically restored the degenerative neurite outgrowth after SAH. Furthermore, inhibition of the expression of p38 also abolished neurites degeneration after SAH. These findings suggested that EGCG promoted neurite outgrowth after SAH via integrin *β*1/FAK/p38 pathway, and the excessive activation of integrin *β*1/FAK/p38 signaling pathway might indicate the severe injury after SAH.

As a result, EGCG increased energy generation, decreased cell injury, and eventually improved neurological function. These results revealed the mechanisms behind the neuroprotective effects of EGCG by inhibition of OxyHb-induced neurite degeneration via integrin *β*1/FAK/p38 signaling pathway and the following cell injury. Therefore, simultaneous inhibition and elimination of neurite degeneration could determine the therapeutic effect of EGCG. Further investigation will be necessary to explore the mechanisms of EGCG transmembrane transport and the ways to improve the bio-availability, which may promote the potential for application to SAH disease.

## Data Availability

Data are available upon request to the corresponding author.
